# Mammals tolerate harmless human presence: Lessons from COVID-19 lockdown on Barro Colorado Island, Panamá

**DOI:** 10.1038/s41598-026-50618-8

**Published:** 2026-06-30

**Authors:** Claudio M. Monteza-Moreno, Jacalyn Giacalone, Mark N. Grote, Daisy H. Dent, Gregory Willis, Margaret C. Crofoot

**Affiliations:** 1 Department for the Ecology of Animal Societies, Max Planck Institute of Animal Behavior, Konstanz, Germany; 2 Smithsonian Tropical Research Institute, Ancon, Republic of Panama; 3 Department of Anthropology, University of California – Davis, Davis, CA USA; 4 Department of Biology, University of Konstanz, Konstanz, Germany; 5 Department of Environmental Systems Science, ETH, Zurich, Switzerland

**Keywords:** Human-wildlife coexistence, Wildlife responses, Human disturbances, Anthropause, Sustainability, Forest management, Ecology, Ecology, Zoology

## Abstract

Human presence in protected forests impacts wildlife, but investigating such impacts is challenging because it is rare to isolate human presence from other anthropogenic factors. The COVID-19 lockdowns in 2020 provided a quasi-natural experiment that reduced human activity on Barro Colorado Island, a tropical forest isolated from most human footprints in Panamá. We used trail-based camera trap data from mammal species to compare a lockdown period (April–July 2020) versus non-lockdown (2019). For all observed species, we tested the hypotheses that human presence impacts activity level for 14 species, and for focal species we also tested diel activity, predator-prey dynamics, group cohesiveness, scent-marking, foraging, and vigilance. To assess lockdown effect, we analyzed our data using negative binomial, logistic and recurrent event analysis, and we contrasted null and alternative models. We also estimated diel activity patterns and used confidence intervals to examine lockdown effects. Based on camera trap observations, human presence on BCI forest was 9 times lower, while 16.2 times lower based on safety book records. Results showed no significant changes in activity level (rate of events) and diel activity for any species; in foraging duration of agouti, collared-peccary, red-brocket deer, and white-nosed coati; in predator-prey dynamic between agouti and ocelot; and in scent-marking of agouti. However, group cohesiveness and vigilance of white-nosed coati and collared-peccary were higher during lockdown. Overall, under lockdown, animal activity and diel activity patterns remained unchanged, although agouti, peccary, coati, and ocelot’s diel activity slightly increased during typical human-active hours. Our results indicate that mammals, living on a managed forest with low anthropogenic impact and disturbance, can tolerate non-consumptive human presence.

**Supplementary Information:**

The online version contains supplementary material available at 10.1038/s41598-026-50618-8.

## Introduction

Human-wildlife interactions shift in space and time, depending on the degree of human-presence and human-footprint at the landscape scale^1,2^. In protected forests, human-wildlife interactions range from non-consumptive practices (e.g., hiking and bird watching) to consumptive practices (e.g., lethal encounters) like illegal or legal hunting^3,4^. Regardless of the nature of the encounters, human presence can alter animal behavior, leading, for example, to increased nocturnality^5^ and/or investment in vigilance^6–8^. Human-presence alone can potentially alter animal behaviors, including mating, seed dispersal and parental care^9,10^.

Because of evolutionary instincts and past experiences, many species respond fearfully towards humans, similarly to how species react to other natural predators. In fact, the risk-disturbance hypothesis suggests that due to body size, humans can elicit the same response as predators^11–13^. Conversely, humans are also hypothesized to shield prey species from predators^6,14^, as prey species tend to become more nocturnal than predators in human-dominated environments^5^. This is possible if species build tolerance towards human-presence, typically as the result of habituation – consistent exposure to stimuli without exerting fear^15,16^.

Understanding the direction of human-presence impact on wildlife is difficult due to lack of controls (e.g., accessible vs inaccessible sites to humans;^17^). In protected forests, humans engage in non-consumptive practices associated with infrastructure (e.g., lodgings, roads and trails), and thus the impacts of human presence can be confounded with a suite of factors ranging from anthropogenic noise (e.g., vehicle and artificial light) to companion of domestic animals^18,19^. Such confounding factors potentially introduce bias, limiting our comprehension of the effects of human-presence alone on wildlife.

Consequently, disagreements exist about whether the effects of human-presence are neutral, positive or negative^20–22^. Understanding the individual and combined effects of human-presence and footprint are critical to conservation management^17^, including minimizing impacts on wildlife while allowing humans to benefit from nature-related activities^23^. Thus far, studies of human-wildlife interactions have focused on activity level and temporal responses, while little is known about behavioral responses (e.g., foraging and vigilance;^24,25^). Measures of animal behavior changes (e.g., diel activity, foraging, vigilance) in response to human-presence alone are important to identify whether or not the responses are detrimental to individual fitness^26,27^. One way to study how human-presence, independent of other anthropogenic factors that tend to accompany human-presence, impacts animal behaviors is through natural experiments that compare systems with and without humans^17^, and without confounding factors like domestic animals, artificial noise or light. The experimental removal of humans at the landscape scale is almost unheard of. However, during restrictions on human activity prompted by COVID-19 in 2020, this is exactly what occurred. The lockdowns resulted in the near absence of humans from many protected areas^17,23^, presenting an opportunity to understand how human-presence impacts animal behavior^17^.

Here, we took advantage of the quasi-natural experiment created by the COVID-19 lockdowns of 2020^17,28^ in Panamá, to compare activity level, diel activity patterns and behavioral responses (e.g., vigilance) of forest mammals, living on Barro Colorado Island (BCI), for 4-months of lockdown and the same period one year earlier. We consider BCI an ideal study site for assessing the response of mammals to human-presence alone, because the management plan for BCI has prevented and controlled other anthropogenic factors (e.g., hunting, logging, habitat disturbance, vehicle traffic, domestic animals) for more than 50 years. Thus, the COVID-19 lockdowns on BCI isolate the impacts of human-presence independent of other associated anthropogenic factors. The comparison before and during lockdown on BCI is strengthened by the fact that there was little difference between climatic conditions between the two contrasted periods (Supplementary Table 1)

We used trail-based camera trap data to test hypotheses (Table 1) about whether or not human-presence impacts mammals’ behavior, when isolated from other anthropogenic disturbance. If human activity alone is perceived as a disturbance by forest mammals, we expect ground-dwelling animals to be especially sensitive because their spatial niche will require the most significant avoidance strategies. Diurnal species may be more impacted by humans than nocturnal species, because human activity is concentrated at times of day when diurnal species are most active. In particular, we hypothesize that human-presence (1) changes activity level and (2) diel activity patterns, (3) shields prey from predators, (4) relaxes group living species’ cohesiveness, (5) disrupts communication signaling, (6) alters foraging behaviors and (7) changes the vigilance of forest mammals living in BCI. We predict that with reduced presence of humans, as measured by camera traps, wildlife will exhibit (1) a higher frequency of visit rates, (2) more activity during day-time, (3) more active predator tracking and prey avoidance, (4) larger party size, (5) higher frequency of scent-marking, (6) longer visit durations when engaged on food search/foraging, and (7) higher levels of vigilance.

**Table 1:** Overview of hypotheses and predictions, and top-level outcome. In our AIC model comparison (Null model - alternative model), when the result is positive the alternative model, including the lockdown effect, is preferred. *This is a WAIC model comparison for models fitted with computational Bayesian simulation^29^.

**Hypothesis**	**Prediction (during lockdowns)**	**Statistical support for prediction**	**AIC difference: null - alternative model**
**H1. Rate of events.** Human-presence affects activity level	**P1.** Higher species monthly rates	No	-12.4
**H2. Diel activity patterns.** Human presence constrains mammal’s temporal activity	**P2.** Mammals’ diel activity increases during hours in which humans are typically most active.	No	Not applicable
**H3. Predator-prey dynamic.** Human presence shields prey from predators	**P3.1** Shorter waiting times of ocelots after agouti detections	No	-1.7
	**P3.2** Longer waiting times of agouti after ocelot detections	No	-1.6
**H4. Scent-marking.** Human presence disrupts species olfactory signaling	**P4.** Odds of agouti scent-marking are higher	No	-1.3
**H5. Group cohesiveness.** Human presence relaxes group living species’ cohesiveness.	**P5.** Party size is higher for peccary and coati	Yes	25.3*
**H6. Engagement on behaviors.** Human presence alters foraging behaviors	**P6.** Species spend more time per visit when traveling and foraging (agouti, peccary, brocket deer, coati)	No	-0.03
**H7. Engagement on behaviors.** Human presence alters vigilance of forest mammals	**P7.** Species odds of vigilance are higher (agouti, peccary, brocket deer, coati)	Yes	4.1

## Methods

### Study site

We collected data on BCI, a 1540 ha hilltop that became an island after the damming of the Chagres river to create Gatun Lake in 1914. BCI is characterized by lowland tropical moist forest, with yearly average temperature of 27^ᵒ^ C and rainfall of 2,631 mm^30–32^; and marked seasonality, where the dry season extends from December through April. BCI’s forests include secondary, undisturbed and old-growth, ranging from 100 years to 400 years old^33^.

The BCI field station was established in 1923 and is managed by the Smithsonian Tropical Research Institute (STRI). Human footprint and impact on BCI landscape are relatively low^34^, with minimal development restricted to laboratories, greenhouses and dormitories that occupy less than 25 hectares, as well as ~40 km of trail network^35^. Trails on BCI are typically not wider than one meter. Because access to BCI is managed by STRI, human-presence on BCI forest is relatively low, consisting of a community of researchers of no more than 80 people (60 residents plus ~20 daily commuters), and staff and tourists. However, the presence of staff is limited to the laboratories area and a maximum of 10 tourists during the week, and no more than 30 during weekends, are limited to the northeastern area of the island, covering about ~3 km of trails embedded in 30-35 hectares of forest close to the field station.

The presence of humans on BCI has two peaks: during the dry season (mid-January – late-March) and the Northern Hemisphere academic break (May – August). Researchers can access most trails on BCI, but as a way to reduce human-impact, two areas of reduced access were established in the 1970’s. These areas are situated in the West (~300 ha) and East (~100 ha) of the island, both bordering Gatun Lake.

Poaching on BCI has been non-existent since the 1980s due to intensive year-round patrolling conducted by a team of game guards^36^. Additionally, as part of the field station management, animal feeding and petting have been prohibited since 1978. Nevertheless, habituation, the use of baits, and animal capture for research purposes can take place^37,38^ for limited periods of time, with approval of STRI Animal Care and Use Committee. Thus, our research site, BCI, experiences no impacts and/or activities typically associated with humans (e.g., hunting, logging, habitat disturbance, vehicle traffic, and domestic animals), affording us the opportunity to investigate responses to the reduction in human presence by an exogenous cause (e.g., COVID-19).

### Study species

We studied non-volant mammals known to consistently occur on BCI^39,40^. The mammal species varied in size, diet and lifestyle. For instance, species size ranges from the spiny pocket mouse (*Heteromys desmarestianus; 0.05-0.09 kg*) to Baird’s tapir (*Tapirus bairdii; 150-300 kg*), although most species included in this study are medium-large sized (> 2 kg, < 15 kg) and three are very large (> 15 kg)^41^. Most mammal species on BCI are frugivorous/omnivorous and frugivorous/granivorous, with a few carnivorous species. With no detections of jaguars (*Panthera onca*) and pumas (*Puma concolor*) in years, nowadays, the predator community of BCI is virtually reduced to ocelot (*Leopardus pardalis*) and jaguarundi (*Herpailurus yagouroundi*). This is because since 1983, there have only been four records of jaguars, with the last one being in 2009. Whereas for pumas, between 1993 and 2015, the presence of this large feline has been confirmed in about 10 non-continuous years, with frequent reports between 1999 and 2001^42^.

Mammals on BCI have diverse lifestyles, including nocturnal, diurnal and crepuscular species. Of the ground-dwelling mammals on BCI, there are only two group-living species (white-nosed coati; *Nasua narica*, and collared-peccary; *Pecari tajacu*, hereafter coati and peccary, respectively), while the rest have a predominantly solitary lifestyle. Most mammals on BCI are relatively common and are broadly representative of forest specialists from Central Panamá^43^. The two most common species are the Central American agouti (*Dasyprocta punctata*, hereafter agouti) and the peccary^40^, whereas the most common predator is the ocelot^44,45^.

Results from other sites suggest that many of the mammals living on BCI exhibit shifts in behavior and activity level in response to human disturbance^5^. However, results are mixed even within the same species. For instance, while Cruz et al^46^, in a comparison of continuous forest vs. fragments and plantations, reported that ocelots temporally avoid humans, Kolowski & Alonso^47^ found that the capture rate and activity patterns of ocelots were not affected by human-presence nor by noise during seismic exploration on an oil concession.

### Camera-trap surveys

We used non-baited camera traps from the BCI Mammal long-term monitoring study. Our cameras were mounted on trees along BCI trails at about 40 cm from the ground and facing the trail path, with no specific view target, and with an average interspacing of 0.7-km (min / max interspace). Cameras were motion-sensored (Reconyx PC900 Hyperfire – Reconyx, Inc, WI, USA) allowing the camera to be triggered when animals pass in front of it. Cameras were set to take three images upon each trigger, with no delay between triggers so that if animals remain active in front of the camera, it accumulates continuous triggers. In our data, an event is defined by triggers accumulated with a cut-off time of 120 seconds.

Our cameras typically receive maintenance (e.g., cleaning, new SD cards and batteries) twice a year (February and August). However, in The Republic of Panamá, the COVID-19 induced lockdowns were officially declared on March 17, 2020 (Panamanian Ministry of Health, 2020). Due to this lockdown in Panamá, no camera servicing occurred in August 2020 and cameras remained in the field until February 2021, when institutional permission was provided for camera maintenance. Then, for all our (formal) analyses, we focused on the contrast between non-lockdown and lockdown conditions, using data from April through July of 2019, compared to the same 4-month interval in 2020, to assess mammals’ responses to the lockdown period in Panamá. Although official lockdowns were declared on March 17, relocation of scientific staff from BCI took about 10 days. Therefore, for our formal analyses, we compared data collected 1 April - 31 July 2019 (hereafter non-lockdown) versus 1 April - 31 July 2020 (lockdown).

During lockdown, only a handful of long-term projects were institutionally allowed to maintain data collection with reduced operation (e.g., < 3 people per project at the time).

We surveyed a total of 20 camera sites during non-lockdown and 15 during lockdown, yielding a sampling effort of 2,050 and 1,487 camera trapping days, respectively. Fourteen of the total deployed cameras were paired, collecting data from the same location in both years. Five cameras stopped operating prior to the end of data collection (July 31 2020). For our analysis, we included species with > 1 observation per year, leading us to exclude two species (*Hydrochoerus isthmius;* capybara and *Dasypus novemcinctus;* nine-banded long-nosed armadillo).

We processed and annotated camera trap images on the free, custom-made image processing and archiving system, Agouti (www.agouti.eu;^48^). We conducted a second review of the photos to annotate counts of key behaviors for focal species, and to extract the exact observed visit duration in seconds from each event. For this second revision, we focused on a subset of four species (agouti, peccary, coati, and *Mazama temama* (Central American red brocket deer, hereafter brocket deer)) using data from 7-paired camera traps.

### Data analysis

**Fig. 1:**
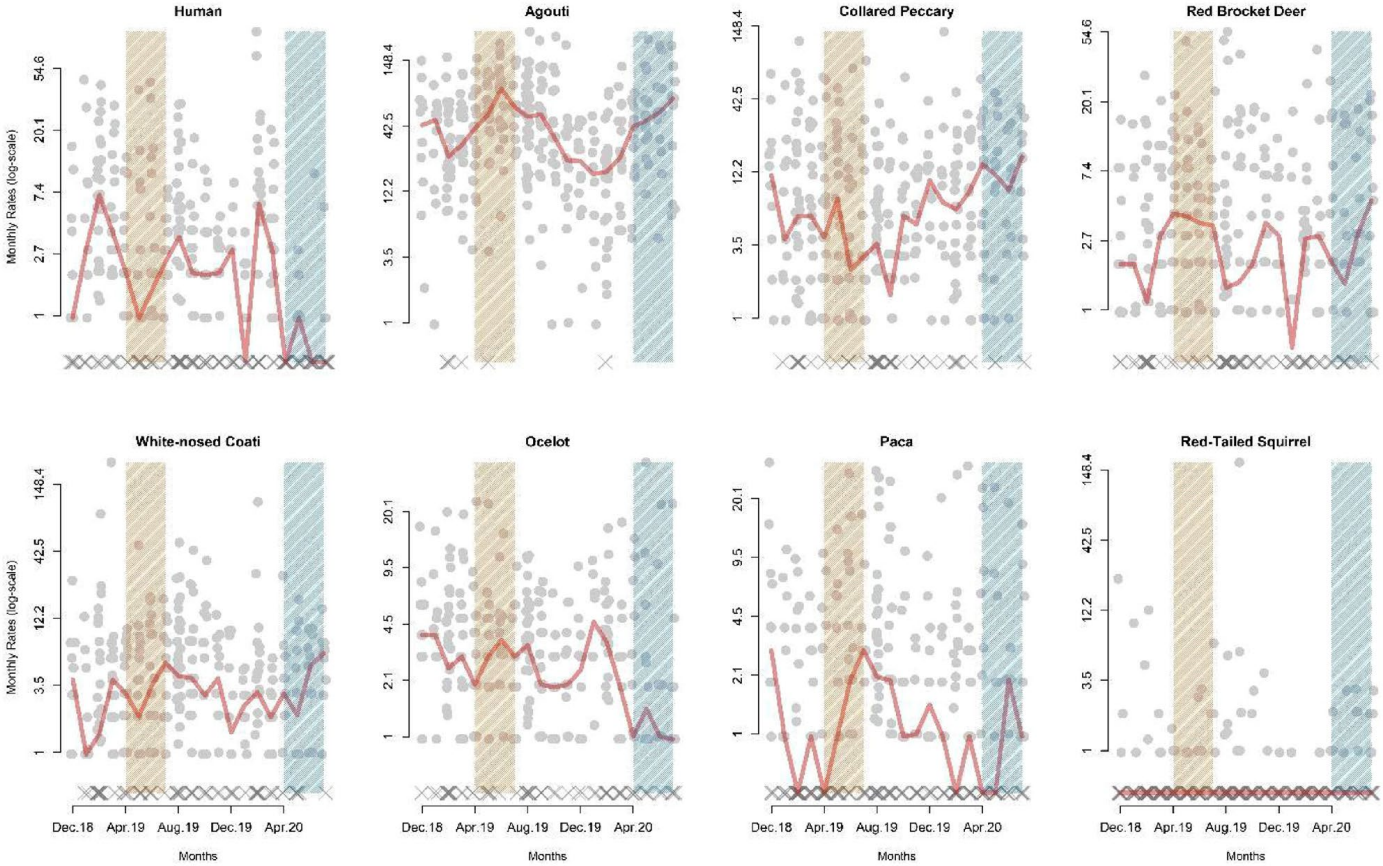
Empirical estimates of monthly rates (log-scale) for Human and seven mammal species, from December 2018 until July 2021. Color-coding shows the four focal months during non-lockdown (orange) and lockdown (blue). The red curve represents the median and grey dots the estimates per camera trap, and x-mark symbols exposes zero observation of a given species at a given camera trap station.

The data were from a quasi-natural experiment created by the COVID-19 induced lockdown^49^, an exogenous factor that largely removed humans from the BCI landscape, which allowed us to shed light on the effects of human-presence on wildlife^17^. To inspect potential seasonal variation among species, we first used the nine species with greatest numbers of observations to display a descriptive monthly-rate of mammal events (Fig. 1) from December 2018 through July 2020. We adjusted the rates to account for partial months of operation at the beginnings and ends of camera deployments. We also produced descriptive estimates for each species for non-lockdown and lockdown (Table 2 and Supplementary Table 2).

Then, to formally evaluate the animal response to lockdown, similar to Tucker et al.^50^, we compared four months (April, May, June and July) of the lockdown period in Panamá of 2020 versus an equivalent non-lockdown period from 2019. Our interest was to compare seven types of responses, including activity level, diel activity patterns and behavioral responses. Specifically, our measure for activity level was (1) the monthly rate of events for 14 forest mammals; our temporal responses included (2) the diel activity pattern of five focal species and (3) the waiting time between prey and predator species; and our behavioral response measures were (4) the odds of scent-marking of territory of the most common species on the island (agouti), (5) the party size of the two group-living species (coati and peccary), (6) the time engaged in foraging and traveling by focal species, and (7) the odds of vigilance of focal species.

#### Model comparisons

Our models aim to assess the responses to human presence of different animal species, each with their own species-specific behavioral and ecological characteristics. To test our predictions about lockdown effects (Table 1), while avoiding multiple species-specific comparisons^51,52^, for each test we fitted a null model with no interaction (between species and period), excepting the (non-parametric) diel activity patterns, and an alternative model including the period effect. We then compared the Akaike Information Criterion (AIC) values for likelihood models^53^ and the Widely Applicable Information Criterion (WAIC) for the Bayesian model^54^. Finally, for each model, we also inspected the goodness of fit using Pearson residuals.

We performed both informal data inspection and formal analyses using packages available in the R Statistical Language – R Core Team, 2021.

#### Activity level

To investigate whether or not human-presence changes mammals’ activity level, we modeled the number of species’ *events* for periods (non-lockdown and lockdown) using a negative binomial mixed model, following the method available in the library *glmmTMB* (Generalized Linear Mixed Models using Template Model Builder) version 1.1.9^55^. In our model, we allowed for an interaction between species and periods, treated the length of the restricted sampling period as an offset and used camera deployments as varying intercepts (“random effects”). The inclusion of the offset allows the model to be interpreted through the metric *rate of events*. We estimated the mean *rate of events* per month and generated 95% confidence intervals per species and period from the model.

#### Diel activity patterns

To assess if human-presence changes mammals’ *diel activity* patterns, we converted the timestamps of events from human and five focal species (agouti, peccary, coati, ocelot, and brocket deer) to solar time, to compare their timing of *diel activity* during non-lockdown and lockdown. For this, we used the methods available in the Activity Pattern package version 1.3.4^56^. We opted to assess the *diel activity* of species for which we obtained more than 50 observations per period to minimize bias^57^. We produced density curves comparing between periods for the same species with 95% confidence intervals.

#### Predator-prey interactions

To test if human-presence shields prey from predators, we applied recurrent event analysis^58^ to the time intervals (*waiting times*) between visits of the most common prey (agouti) and predator (ocelot) on BCI. We fitted a piece-wise exponential additive mixed model from the pammtools package version 0.7.3^59^, to estimate the rate of occurrence of agouti visits after an ocelot visit as a smooth function of time, and analogously ocelot visits after an agouti visit. The model used a time horizon of 3-days and is adjusted for unique (“random”) effects of camera deployments. We compared the prey-predator *waiting times*^60^ during non-lockdown versus lockdown, as a measure of active avoidance and tracking^61^. Here, the agouti *waiting time* refers to the time it takes to observe an agouti after the visit of an ocelot, and ocelot *waiting time* to the time it takes to observe an ocelot after the visit of an agouti. Finally, we produced plots of the occurrence rates over time for both species: the *waiting time* for an ocelot event after an agouti visited the location and vice versa.

#### Group living species’ cohesiveness

We assessed whether or not human-presence relaxes group living species’ cohesiveness, by comparing *party sizes* between non-lockdown and lockdown of two group living species occurring on BCI, peccary and coati. We defined *party size* as the number of individuals captured per (camera trap) event – a proxy for spatial group cohesiveness^62–64^, which has been found to be correlated to predation risk^65,66^. The peccary is a species known to live and move in groups typically ranging from 1 to about 15 individuals^67,68^, including members of both sexes and all ages. Conversely, in coati, adult males are known to be solitary for a large portion of their lifetime, and groups, largely composed of females and juveniles, experience fission-fusion dynamics^62,69^. Consequently, for coati, we naturally expect many occurrences where the *party size* is one, with the modal and the median *party size* also being one, in either non-lockdown or lockdown. Additionally, camera traps might inherently fail to detect all group members because, for example, half of the group might pass behind the camera trap^70^. *Party size* is therefore a proxy for group cohesiveness. Along with potential changes in mean *party size*, we pay attention to the upper range of *party size*.

We fitted a multilevel negative binomial model for *party size*, using the *brms* (Bayesian Regression Models using ‘Stan’) package version 2.21.0^71^. We allowed for an interaction between species and periods, and acknowledged that *party size* is necessarily greater than zero, fitting a truncated form of the distribution. We treated camera sites as varying intercepts and ran the model using four chains, four cores and 2000 iterations.

#### Scent-marking

To test whether or not human-presence disrupts communication signaling, we modeled the binary variable indicating agouti’s *odds of scent-marking* to compare this behavior during non-lockdown and lockdown. Agouti’s scent-marking is a very distinct communication signaling behavior, observed when an agouti individual deposits scent-marks on the forest floor using their glands anterior to the anus^72^. We scored each trigger event as indicating scent-marking or not and fitted a logistic mixed model using the method available in the library *glmmTMB* (version 1.1.8;^73^), where we used camera deployments as varying intercepts.

#### Foraging behaviors

To investigate if human-presence alters time spent on foraging, we used the exact *visit duration* in seconds, coded as traveling or foraging behavior, from four seed dispersers species (agouti, coati, peccary and brocket deer)^74–77^. We classified traveling events as triggers where individuals move in front of the camera’s field of view without engaging in further behaviors, and foraging events where individuals walk with their heads down actively sniffing the floor, or with a visible fruit in their mouth. We then used the *coxph* function of the *survival* library to fit a Cox proportional hazard regression model^78^ for the *visit duration* of behaviors between non-lockdown and lockdown, while allowing for an interaction between species and periods. We adjusted for the clustering of observations within cameras using cluster-robust standard errors.

#### Vigilance

We assessed if human-presence changes mammals’ vigilance, by modeling count data of vigilance behavior for four focal species (agouti, coati, peccary and brocket deer) and comparing non-lockdown and lockdown periods. Similar to Tsunoda^79^ and Lima et al^80^, we extracted vigilance events by scoring events as ones when animals moved their heads up as they walked in front of a camera, and zeros when animals walked in front of the camera with heads down. We fitted a logistic mixed model using the method available in the library *glmmTMB* (version 1.1.8;^73^), and used camera deployments as varying intercepts in our model. We summarized the estimates of the *frequency of vigilance* from our model in Table 3B including standard errors.

## Results

Our accumulated sampling effort between both non-lockdown and lockdown was 3,537 camera trap nights, as some deployments were shorter than expected (122 camera trap nights/deployment). In 2019, out of 20 camera traps, six camera traps stopped operating prior to July 31, whereas in 2020, this was true for five out of 15 camera traps. Thus, our sampling effort per period was 2,045 and 1,488 camera trap nights, corresponding to non-lockdown and lockdown, respectively.

Based on our trail camera traps, human-presence on BCI was nine times lower during lockdown. Human events went from 271 events during non-lockdown to 29 during lockdown, with a median empirical monthly rate of 1.7 and 0.2, respectively (Supplementary Table 2 and Fig. 1). Based on safety records maintained by the BCI administration, between April and July of 2019, the number of people that visited BCI forest was 665, whereas for 2020, for the same period of time, it was 41. This is 16.2 times less people in BCI forest.

For mammals, the empirical mean *rates of events* for species differed mildly between non-lockdown and lockdown (Supplementary Table 2 and Fig. 1). The species that exhibited a relative increase in their empirical mean *rates of events* between non-lockdown and lockdown were Baird’s tapir (0.1 vs. 0.2), red-tailed squirrel (0.4 vs. 0.6) and white-faced capuchin (0.2 vs. 0.3). The empirical mean *rate of events* of the two most common species on BCI only exhibited a mild increase. From non-lockdown to lockdown, the empirical mean *rate of events* for peccary changed from 13 to 17, whereas for agouti it changed from 61 to 59 (Supplementary Table 2). Our longitudinal monthly-rate of events, from December 2018 to July 2020, shows the monthly (seasonal) variation in rates for all species (Fig. 1).

**Table 2:** Empirical observations and mean values of model estimates for rate of events, for a sampling effort of 2045 camera traps days during non-lockdown and 1488 during lockdown.

Species	Empirical observations		Modeled estimates (mean)	
	Non-lockdown	Lockdown	Non-Lockdown	Lockdown
Humans	271	29	—	—
Agouti	4403	3121	56.9 (45.3 – 71.3)	49.2 (38.4 – 63.2)
Collared-peccary	834	760	7.4 (5.1 – 10.7)	10.3 (7.1 – 15.1)
Brocket deer	346	276	5.2 (3.5 – 7.9)	4.4 (2.7 – 7.1)
White-nosed coati	311	236	5.2 (3.5 – 7.8)	5.1 (3.2 – 8.0)
Ocelot	274	197	4.9 (3.3 – 7.4)	3.2 (1.9 – 5.3)
Paca	194	171	3.4 (2.1 – 5.4)	2.6 (1.5 – 4.5)
Common opossum	68	40	1.7 (1.0 – 2.9)	1.1 (0.6 – 2.3)
Red-tailed squirrel	26	31	0.8 (0.4 – 1.7)	1.4 (0.8 – 2.7)
White-faced capuchin	15	14	0.7 (0.3 – 1.4)	0.6 (0.2 – 1.4)
Spiny rat	12	10	0.5 (0.2 – 1.2)	0.4 (0.2 – 1.2)
Baird’s tapir	5	15	0.2 (0.1 – 0.8)	0.4 (0.2 – 1.2)
Jaguarundi	13	1	0.6 (0.3 – 1.3)	0.1 (0.0 – 0.7)
Northern tamandua	7	5	0.5 (0.2 – 1.1)	0.4 (0.1 – 1.1)
Spiny pocket mouse	3	2	0.1 (0.0 – 0.6)	0.2 (0.0 – 0.8)

Overall, based on the AIC contrast between the null model and an alternative model with lockdown effect, the evidence for lockdown effects is relatively weak for *rate of events*, agouti’s *odds of scent-marking*, and *visit duration* of four focal species when engaged in foraging behaviors. We found no evidence for active predator tracking (from ocelot to agouti) or for active avoidance (from agouti to ocelot). We found support for increased *frequency of vigilance* during lockdown for one out of four focal mammal species. We found support for the lockdown effect on group cohesiveness in group living species on BCI, with larger groups recorded during lockdown. Table 1 summarizes the output of our contrast between null and alternative models.

### Activity level

**Fig. 2:**
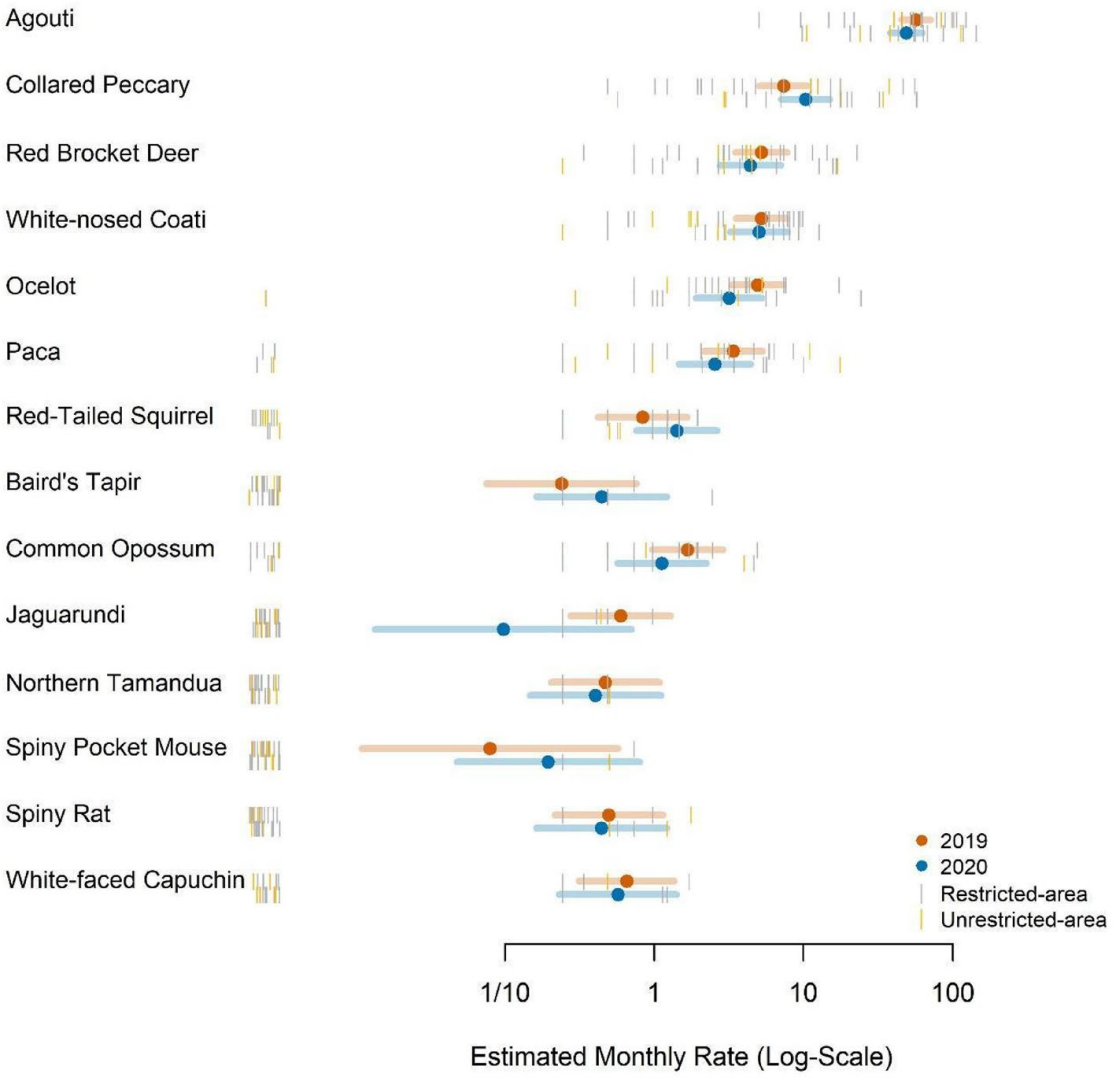
Negative binomial glmmTMB model estimates for mammal species on Barro Colorado Island for a four-month non-lockdown period in 2019 (orange) and lockdown period of 2020 (blue). Empirical observations per camera trap station (vertical tick marks) are shown jittered and color-coded based on the management of BCI (restricted versus unrestricted area for research). Vertical tick marks next to species names represent camera traps with zero observations. Horizontal lines give the 95% confidence interval and the dot shows the estimated mean of monthly rate per species and period. Noteworthy that white-nosed coati and Northern tamandua represent semi-arboreal species, and red-tailed squirrel and white-faced capuchin predominantly arboreal.

Overall, we found no support for changes in activity level when contrasting non-lockdown and lockdown, as the frequency of *rate of events*, measured from a negative binomial mixed model (*glmmTMB*), was not significantly higher during lockdown. During lockdown, the *rate of events* showed a tendency to increase in four species (peccary, red-tailed squirrel, Baird’s tapir, and spiny pocket mouse). While for the rest of the species, the *rate of events* decreased (Table 2 and Fig. 2).

During non-lockdown, nine out of the 14 mammal species had no events on at least one deployment. Whereas, during lockdown, 10 species had zero events on at least one deployment (ocelot, paca, red-tailed squirrel, Baird’s tapir, common opossum, jaguarundi, northern tamandua, spiny pocket mouse, spiny rat, and white-faced capuchin). The confidence intervals for lockdown and non-lockdown rate of events overlap for all 14-mammal species, informally confirming the lack of support for a lockdown effect (Fig. 2). The statistical summary of our model estimated *rate of events* for all species is given in Table 2.

### Diel activity patterns

**Fig. 3:**
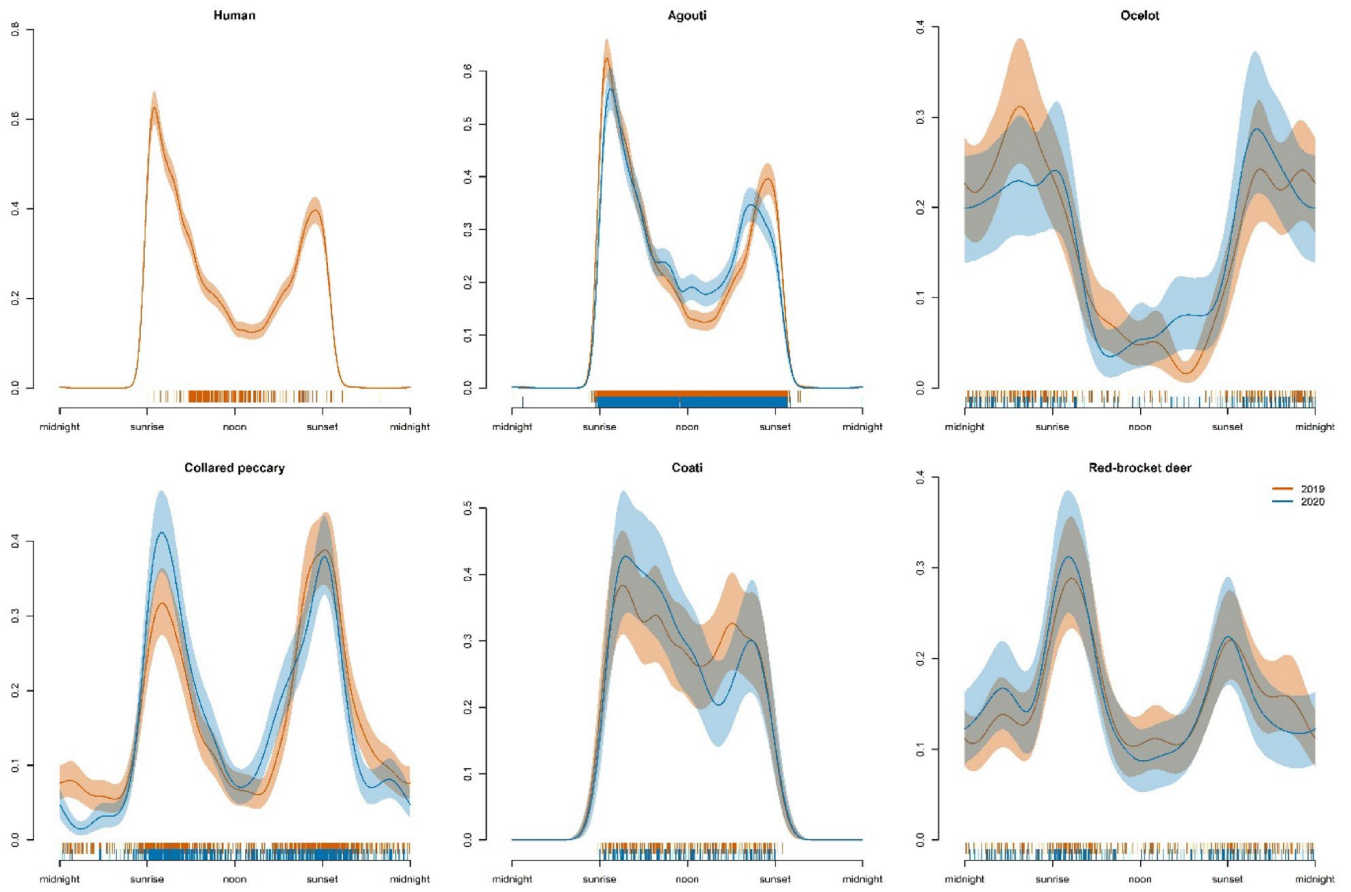
Kernel density curves, with 95% confidence interval bands from 10K reps, contrasting *diel activity patterns* during a non-lockdown period (orange) versus lockdown (blue) for Human (N = 271 during non-lockdown vs N = 29 during lockdown), and five mammal species including: Central American agouti (N= 4403 vs N= 3121), ocelot (N= 274 vs N=197), peccary (N= 834 vs N= 760), coati (N= 311 vs N= 236) and brocket deer (N= 346 vs N= 276).

The *diel activity* patterns of humans on BCI were less pronounced during lockdown, when compared to non-lockdown where the peak of human activity ranges from 8:00 AM to 3:00 PM. The confidence intervals of human *diel activity* between non-lockdown and lockdown overlapped throughout the day, excepting from mid-morning to midday (Fig. 3).

For all five focal species, the estimated *diel activity* was not different between non-lockdown and lockdown. As informally assessed using the overlap of the confidence intervals, a few short portions of the day differed between non-lockdown and lockdown for agouti, peccary and ocelot. Under lockdown, the *diel activity* for agouti and ocelot tended to be greater from midday towards sunset; for coati and peccary it tended to be greater from dawn hours towards midday; and for brocket deer it tended to be lower from mid-morning until 2:00 PM (Fig. 3). The respective increase in *diel activity* of agouti, ocelot, peccary and coati, as well as the decrease of brocket deer overlaps with hours of the day where humans would typically have high activity (Fig. 3).

### Predator-prey interactions

**Fig. 4:**
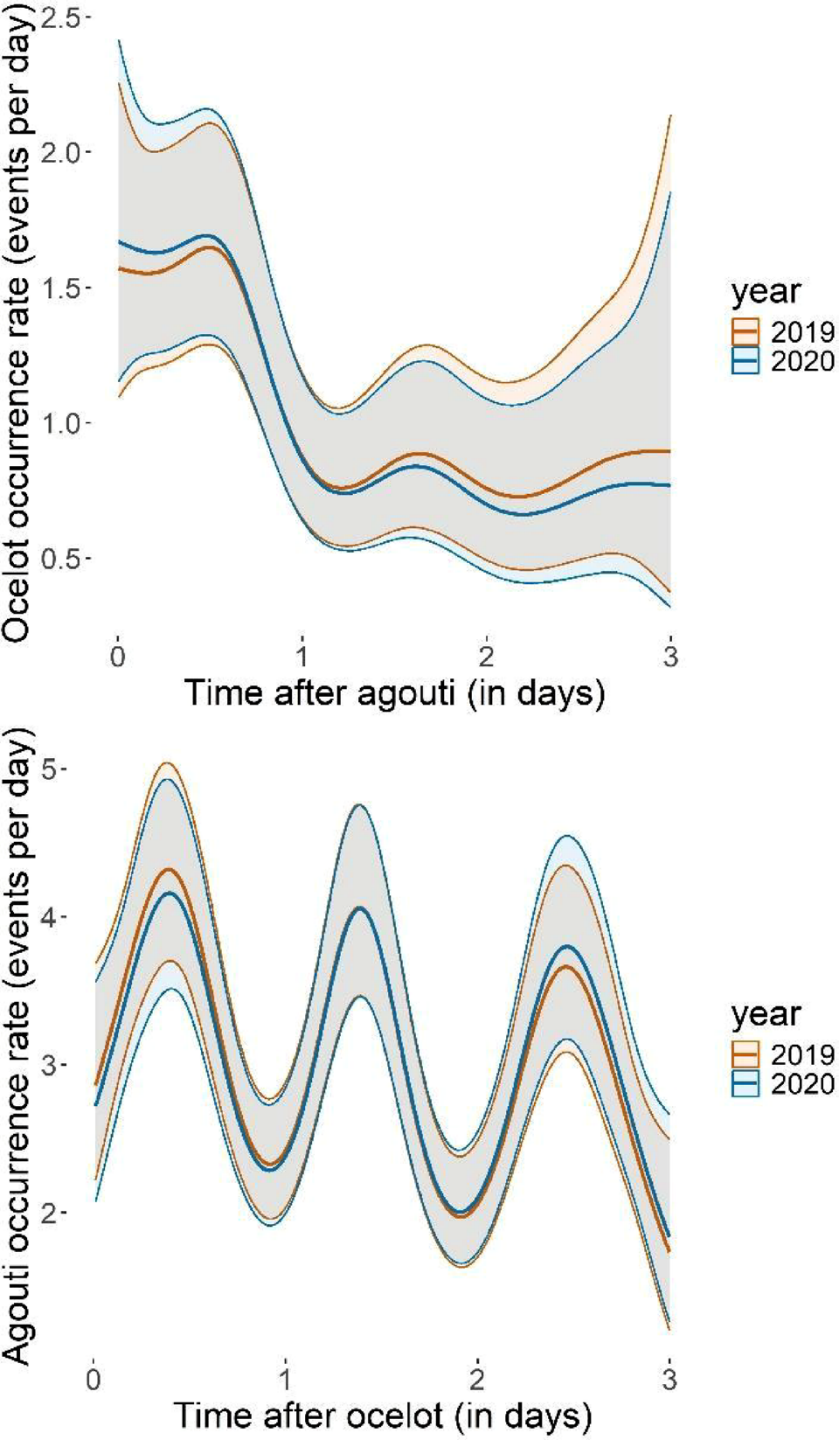
Recurrent event analysis to the time intervals (waiting times) between visits of predator and prey, comparing non-lockdown (orange) and lockdown (blue). Time after agouti: refers to the time until the next ocelot detection after an agouti had arrived first. Time after ocelot: the time until the next agouti detection after an ocelot arrives first. Shaded regions correspond to 95% confidence intervals.

We did not find statistical support for either predator avoidance nor prey tracking during lockdown. The occurrence rates shown in Fig. 4 are broadly similar between lockdown and non-lockdown, and the respective confidence band noticeable overlaps. Although, there is a tendency for a greater intensity of ocelot visits for about a half-day after an agouti occurrence.

### Group living species’ cohesiveness

For both social species, observed *party sizes* were larger during the lockdown, as compared to the non-lockdown. For coati, the empirical mean *party size* was higher during lockdown (mean = 2.0; sd = 2.1) than during non-lockdown (mean = 1.9; sd = 1.6). The range of observed *party size* varied from 1 to 13 individuals during lockdown, and 1 to 10 individuals during non-lockdown. Similarly, the empirical mean *party size* for peccary was higher during lockdown (mean = 2.2; sd = 2.0) than during non-lockdown (mean = 1.9; sd = 1.8). The range of the peccary *party size* was 1 to 15 during lockdown and 1 to 16 during non-lockdown.

**Fig. 5:**
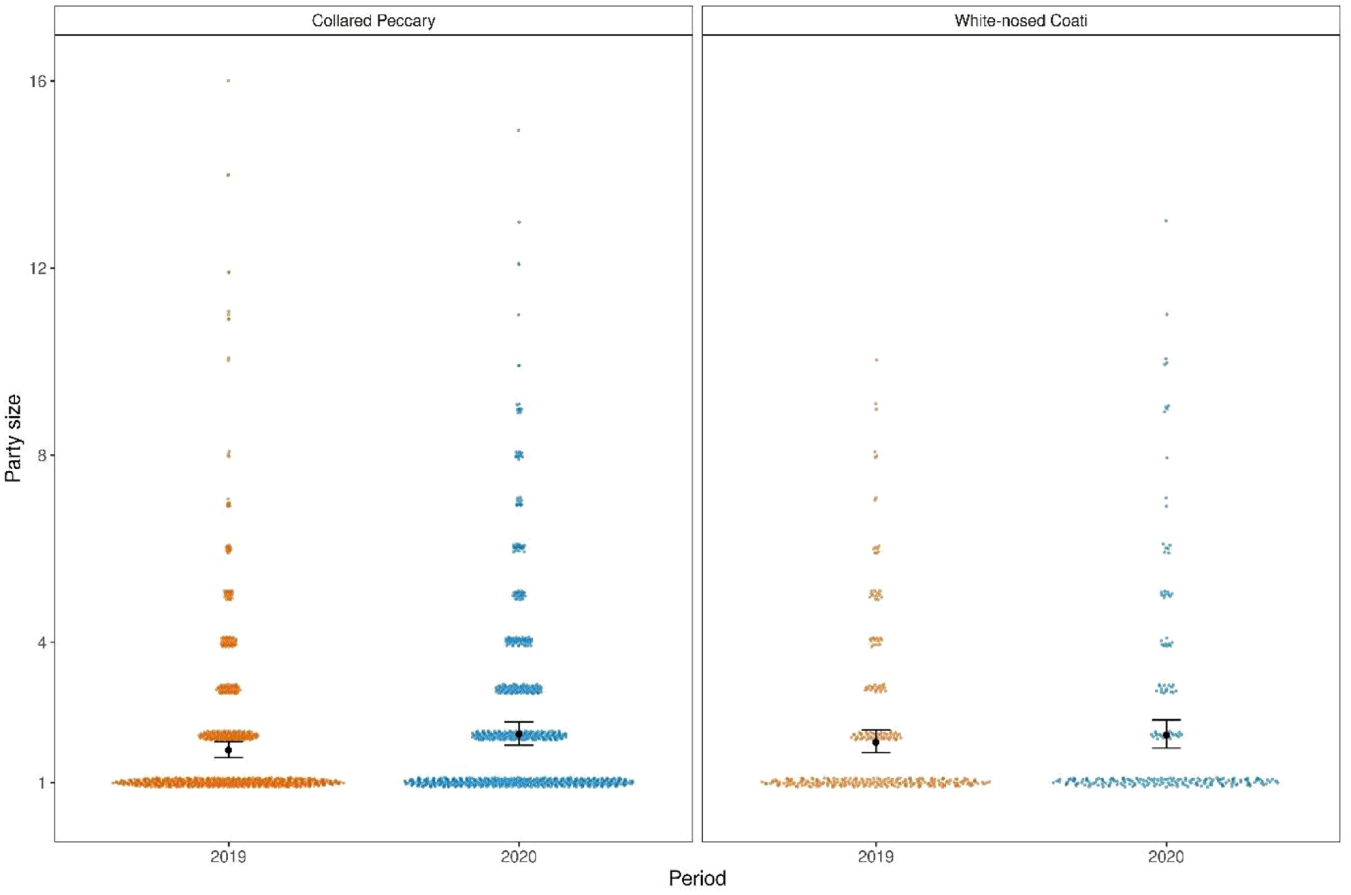
Beeswarm plot showing BRMS model estimates for the party size of two group living species: peccary and coati. Model means with 95% posterior credibility intervals are shown in black. For each species, empirical party size observations are displayed in orange for the non-lockdown period and blue for the lockdown period.

For white-faced coati, during lockdown, our model estimated a mean *party size* of 2.0 (SE = 1.7) individuals per record, while outside lockdown the estimate was 1.8 individuals (SE = 1.5). For either period, the estimated 75^th^ percentile was a *party size* of 2 and the 97.5^th^ percentile was a party of 6 (Fig. 5). For peccary, during lockdown, our model estimated a mean *party size* of 2.0 (SE = 1.7) individuals per record, while outside lockdown the estimate was 1.7 (SE = 1.3). For either period, the estimated 75^th^ percentile was a *party size* of 2, and the 97.5^th^ percentile was 7 during lockdown and only 5 during non-lockdown (Fig. 5).

### Scent-marking

Agouti’s *odds of scent-marking* did not differ between the lockdown vs. non-lockdown. The empirical count of agouti scent-marking during non-lockdown represented 11% of total events and 9% during lockdown. Based on our logistic model, which transforms percentages into log odds, the *odds of scent-marking* when comparing non-lockdown (0.12; CI: 0.07 – 0.18) and lockdown (0.10; CI: 0.05 – 0.22) were very similar.

### Foraging behaviors

For all four focal species, empirical estimates of *visit duration* were longer during the lockdown, as compared to the non-lockdown. While engaged in foraging, the mean *visit duration* for agouti was 24.1 seconds during lockdown vs. 22.9 during non-lockdown; for peccary this was 64.2 vs. 55 seconds; for brocket deer 65.2 vs. 13.5 seconds; and for coati 47.7 vs. 27.7 seconds. Our survival plots (Supplementary Fig. 1) and AIC comparison showed no significant support that animals invest more time foraging during lockdown compared to non-lockdown (Table 3A).

**Table 3:** Percent of events in which an animal of four focal species was engaged in foraging (A) and vigilance (B), during non-lockdown and lockdown. Model estimates of the median visit durations when engaged in foraging (A), with 95% confidence intervals, and of the odds of vigilance (B), with standard errors.

Focal species	Non-lockdown		Lockdown	
Foraging (A)				
	Percent of events	Model estimates	Percent of events	Model estimates
Agouti	28.3	7 (6, 9)	33.6	8 (6, 9)
Peccary	18.5	19 (13, 42)	29.6	23 (15, 45)
Brocket deer	21.1	6 (5, 16)	14.3	16 (11, 25)
Coati	37.5	9 (5, 86)	51.2	16 (13, 18)
Vigilance (B)				
	Percent of events	Model estimates	Percent of events	Model estimates
Agouti	9.2	0.1 (0.02)	9.6	0.1 (0.03)
Peccary	15	0.2 (0.05)	15.8	0.2 (0.05)
Brocket deer	29.2	0.4 (0.11)	46.8	1.1 (0.3)
Coati	1.8	0.02 (0.02)	3.0	0.04 (0.03)

### Vigilance

We found a higher *frequency of vigilance* during lockdown. The empirical frequency of vigilance was higher for all species during lockdown than non-lockdown (agouti, 9.6% vs. 9.2%; peccary, 15.8% vs. 15%; brocket deer, 46.8% vs. 29.2%; and coati, 3% vs. 1.8%) (Table 3B).

Our estimates from a logistic mixed model indicate that the *frequency of vigilance* of brocket deer was three times higher during lockdown compared to non-lockdown, and that *frequency of vigilance* was also modestly higher for the other three species (agouti, peccary and coati) during lockdown than non-lockdown (Table 3B).

## Discussion

In this study, we used the substantial reduction of anthropogenic activity on BCI, prompted by COVID-19, to investigate mammals’ responses to human-presence. To do so, we contrasted mammal activity level and behaviors during April-July of 2020, a period of restriction on human presence, to the same months in 2019, a period of normal human activity on BCI. Our findings provide no support for the hypothesis that human-presence alone impacts mammals’ behaviors. We did not find statistical support for changes in activity level, diel activity, predator-prey dynamic, scent-marking and visit durations when foraging. However, we found a lockdown effect for two behavioral measures (group cohesiveness and vigilance). Our results highlight a minimal response of mammals to human presence in a protected forest, with minimal human-footprint, suggesting mammals might overcome trade-offs associated with human-presence alone when such presence is harmless or non-consumptive.

### Activity level

Our empirical estimates show signs of seasonal fluctuation for humans and species of mammals on BCI (Figure 1). We found that when humans were largely removed from BCI, during COVID-19 induced lockdowns, human-presence on BCI was nine times lower. Our observations are from a trail-based network of camera traps distributed throughout the BCI landscape, therefore we captured a sound representation of human-presence at the landscape level.

We found weaker evidence for the hypothesis of lockdown effects on activity level, compared to the null hypothesis of no lockdown effects. The direction of the response to lockdown and reduced human presence varied by species. However, ten of the 14 species studied (agouti, brocket deer, coati, ocelot, paca, common opossum, jaguarundi, northern tamandua, spiny rat and white-faced capuchin) had lower *rates of events* during the lockdown, although the magnitude of the effect varied substantially, directly contradicting the hypothesis that human presence disrupts mammal activity level. Alternatively, our results might also indicate that mammals on BCI ceased responding to human presence, as proposed by Higham and Shelton^81^. Considering mammals on BCI do not interact with humans outside of scientific contexts (e.g., no feeding, hunting occurs), and thus may habituate to their presence. Similarly, Cruz-Díaz et al^82^ reported habituation to tourists in Corcovado National Park, Costa Rica, as animals did not change activity in response to lockdown.

We predicted higher activity by species with daytime activity, however the most common diurnal species (agouti) on BCI exhibited the greatest decrease *rate of events* during lockdowns. Other diurnal species (coati and capuchin) and cathemeral species (brocket deer, ocelot, jaguarundi and tamandua) also experience a decrease in activity. One possible reason for this response is that if humans shield these two species from predators^6^, in the “absence” of this shield species may be less active. Three nocturnal (paca, common opossum and spiny rat) also decreased activity, although humans are less active in BCI’s forest during nighttime.

When evaluating data from 102 projects from around the world, Burton et al^23^ also identified divergent patterns in mammal responses. They reported a decrease in carnivore activity as human activity increased. Conversely, in our study site, two carnivore species (ocelot and jaguarundi) showed decrease in activity during the COVID-19 lockdowns, when human presence on BCI decreased 9-fold. If humans scare away predators, these two species should have exhibited higher activity when humans were much less present on BCI. Of the diurnal species, only one species experienced a mild increase in activity, the red-tailed squirrel. The highest increase in activity occurred in the cathemeral peccary, a species known to be very shy around humans.

The direction of the response based on strata use was also mixed among species. Of the 14 species, nine are ground-dwelling mammals, three are semi-arboreal and two are predominantly arboreal. Of these, three of the ground-dwelling mammals (peccary, Baird’s tapir, spiny pocket mouse) responded positively to the reduction of human-presence on BCI; all three semi-arboreal species (coati, common opossum and tamandua) responded negatively; and of the predominantly arboreal species, one responded positively (red-tailed squirrel) and one negatively (white-faced capuchin). While forest floor camera trapping might not capture a human-presence effect on predominantly arboreal species, the higher monthly *rate of events* of the red-tailed squirrel suggests this species may be exploiting the forest floor much less in the presence of humans. Conversely, capuchins might be constrained in their exploitation of forest floor, as ground-dwelling predators would be more common with lower human presence^14^.

### Diel activity patterns

During a typical year of human presence at BCI, human *diel activity* patterns are highly concentrated from approximately 8 AM to 3 PM. This pattern overlaps with working hours when most researchers access the BCI forest, with some activity earlier and later corresponding to researchers temporarily staying/living on BCI due to the nature of their research.

A global meta-analysis by Gaynor et al^5^ reported human presence altered animal *diel activity*. More recently, a study using contemporary data from the COVID-19 lockdown, reported increased nocturnality in relation to higher human activity^23^. However, the data associated with these findings came from scenarios where humans co-occurred with different factors (e.g., domestic animals, distance to human dominated landscapes). While our results do not suggest increased nocturnality, we found that when human-presence was reduced during lockdown, morning activity increased in peccary and coati, midday activity increased in agouti and afternoon activity increased in ocelot. These increases in animal *diel activity* took place at times of day when human activity would typically have been greater in non-lockdown conditions. However, similar to Cruz-Díaz et al^82^ and Béllo Carvalho et al^83^, our results suggest that when comparing non-lockdown vs. lockdown from scenarios measuring human-presence alone, overall *diel activity* patterns were not different for any species. Instead all three studies show variation in intensity of diel activity at the hour-level. These results may, in turn, imply that the stimuli produced by non-consumptive human-presence might not trigger a response from mammal species that influences their diel activity patterns.

Interestingly, Burton et al^23^ reported increased nocturnality in carnivores in relation to increased landscape modification, including higher human activity. In our study, we found no significant changes in the overall *diel activity* pattern for ocelot, which is the main carnivore species on BCI. We do observe, however, more pronounced nocturnal activity during non-lockdown, when humans were more active on BCI trails (Fig. 3).

### Predator-prey interactions

When exposed to reduced human-presence, prey avoidance of predators was not more pronounced, contradicting the human-shield hypothesis^6^. And, although a transient increase in ocelot occurrences can be seen during lockdown, this feature is not statistically supported, also contradicting the human-shield hypothesis. Swinkels et al^61^, who also evaluated prey tracking and predator avoidance in BCI, reported different results than our study, as their study provided evidence for longer time between a prey animal visit after an ocelot had passed and shorter time for an ocelot to visit after a prey had passed than by chance. However, in their study, camera traps were deployed off-trails, where detection probability is potentially higher for most species^84^, excepting carnivores which are more likely to be captured on trails^85^.

### Group living species’ cohesiveness

The two group living species that we studied to assess group cohesiveness, peccary and coati, are known to be secondary prey items of ocelot after agoutis^86,87^. Thus, our findings of higher cohesion during lockdown may suggest that when human-presence is largely reduced, group living species might need to travel more cohesively to potentially minimize predation risk^88^. These results support the hypothesis that human-presence shields prey species from predators^6^, as when there is frequent presence of humans, group living species can afford to travel more dispersedly across the landscape. This is either because human-presence is disrupting group cohesion and/or because human-presence influences the dynamic between group living species and their predators.

Our results indicating higher cohesion warrant cautious interpretation as most of our party sizes were just 1-3 individuals for peccary, and 1 for coati (Fig. 5). We argue this is because camera trap data experience two inherent issues: sampling method and in the case of coati, life history. For either group living species sampling party size with camera traps can be limiting because by chance we may only capture one individual at a time, as they can be travelling spaced far apart. Also, the camera has a limited range of view and most individuals, even if traveling cohesively, could be traveling behind the camera traps^69^. For coati, we may have captured solo males and partial groups, because males typically travel solo and coati experience fusion-fission and are semi-arboreal^63,69^. Nevertheless, despite these caveats, the contrast between lockdown and non-lockdown in the upper tail of the distribution (the 97.5^th^ percentile) for both group living species is notable (WAIC = 25.3, Table 1 and Figure 5).

Previous studies have demonstrated a correlation between predator risk and party size^66^. It has also been reported that ingroup cohesion increases when predation risk is higher (e.g., for the *Lama guanicoe*^89^;). Group size, and potentially group cohesion, have implications for the investment in antipredator behaviors^90^ and consequently feeding behaviors.

It is also possible that if humans are perceived as predators by peccary and coati, the predator stimuli exerted by humans to these two species would be absent during lockdown. And that also, due to their body sizes^91^ ocelots (mean body mass: 11.1 kg) do not trigger sufficient fear in peccary (19.8 kg) and coati (4.3 kg) to influence group cohesion. In such a case, an alternative explanation for higher *party size* during lockdown is that in lower presence of a perceived predator, greater competition between groups could induce higher ingroup cohesion^92^.

### Scent-marking

When contrasting a period of substantial reduction of human-presence versus a typical period of human-presence, we found no differences in the *odds of scent-marking* by agouti, suggesting that human-presence does not disrupt chemical communication, via glandular secretions, occurring on agouti at BCI. Considering scent-marking is a strategy used by agoutis to defend resources^72,93^, which is also associated with seed dispersal^94,95^, our results also indicate that human-presence alone is unlikely to alter cascading consequences of animal behaviors such as seed dispersal, an important ecological service^96^.

Our study period (April to July) overlapped with the peak of fruit production of a common palm on BCI, *Astrocaryum standleyanum*^97^, which agouti caches for later consumption during the rainy season^98^ when fruit availability in the forest is low^31^. Thus, it is also possible that scent-marking occurs infrequently during our sampling period due to high food availability and the likelihood that human-presence alters such behaviors is inherently low.

### Foraging behaviors

When human presence was reduced during lockdown, the overall *visit duration* of four mammal’s species (agouti, coati, peccary and brocket deer) was not higher than when humans had greater presence on BCI, based on survival plots and AIC comparison. However, while our overall results are not statistically significant, it is worth noting that brocket deer and coati engaged five and 1.7 times more on foraging during lockdown, respectively, based on our empirical estimates (Table 3A). Although these four species forages while moving through the forest, it is possible that targeting focal fruiting trees could have exhibited different results. A study conducted by Osugi et al^99^, in which they used focal fruiting tree species, showed that raccoon dogs (*Meles anakuma*) and japanese badgers (*Nyctereutes procyonoides*) shifted to more diurnal foraging engagement and increased foraging duration, when human presence declined as a result of COVID-19 lockdown.

Triay-Limonta et al^100^ hypothesized that human disturbance negatively impacts the role of frugivore mammals in seed dispersal mechanisms, but found no support in a review of 275 articles as no study incorporated anthropogenic disturbance in their metrics when assessing the effectiveness of seed dispersal. However, evidence exists that human presence or disturbance affects activity level by frugivore species (e.g., brocket deer and collared peccary^101^;), potentially resulting in cascading effects like affecting the effectiveness of seed dispersal. Although our results do not directly measure the effectiveness of seed dispersal, the empirical estimates for brocket deer warrants attention, because if frugivores engaged less in foraging behaviors in the presence of humans, it may have consequences on forest structure dynamics.

### Vigilance

Our findings about vigilance are interesting because during substantial reduction of human-presence, the *frequency of vigilance* increased overall. If humans are less present in the landscape, animals tend to be more vigilant, potentially because of higher predation risk, supporting at first glance the hypothesis that human-presence shields prey species from predators^6^. Similarly, Ortiz-Jimenez^102^ reported that, during COVID-19 lockdowns, California ground squirrels (*Otospermophilus beecheyi*) increased vigilance towards aerial predators in association to a shift of spatial concentration of humans accompanied by dogs. Previous studies showed similar patterns of lower vigilance by antelopes in the presence of humans near settlements^103^.

However, comparing the influence asserted by humans as predator shields, versus the direct predation risk imposed by humans on other mammal species, is difficult. In our results, the *frequency of vigilance* during lockdown was higher for brocket deer; for coati, modestly higher; and for agouti and peccary, remained the same (Table 3B). The fact that the most common prey item (agouti) of the main predator in BCI, ocelot, did not change vigilance between lockdown and non-lockdown contradicts the shield hypothesis^6^. One could expect that humans influence vigilance more in smaller species, but we found no clear pattern to assert this claim. In fact, an increase of vigilance has been reported in large mammals such as elk, which exhibited higher vigilance towards the presence of hikers/hunters^104^, and giraffes, which showed higher vigilance towards the direct presence of researchers.

Overall, our findings shed light on the effects that human-presence represents to non-volant mammal species of BCI. Our results show that the management of BCI, which excludes anthropogenic disturbances (e.g., traffic, hunting, logging, domesticated animals) while controlling the number of humans accessing the BCI forests, does not alter activity level, diel pattern, predator-prey dynamic, scent-marking and foraging engagement. However, our results indicate that group cohesiveness and frequency of vigilance might be influenced by human-presence. Our results differ from previous studies, suggesting mammal species benefit from human presence while others experience drawbacks. Instead, our results indicate that in forests, with minimal human footprint and managed human access, mammals do tolerate human presence. Our study also differs from many COVID-19 lockdown studies by focusing on the effect of human presence per se in a system where common confounding factors (e.g., dogs, 102) are largely absent. This makes our before-during lockdown comparison particularly informative for isolating the role of human presence itself.

## Supplementary Information

Supplementary Information.

## Data Availability

The data and scripts used in this work will be available via Mendeley Data upon peer-review process.
