# Supplementary material for: Mammals tolerate harmless human presence: Lessons from COVID-19 lockdown on Barro Colorado Island, Panamá
Source: Sci Rep. 2026 Jun 30;16:19927. doi: 10.1038/s41598-026-50618-8 (PMC13319469; doi:10.1038/s41598-026-50618-8)
Supplement: Supplementary file 1 — Supplementary Information. [file 41598_2026_50618_MOESM1_ESM.docx]

**Supplementary Information**


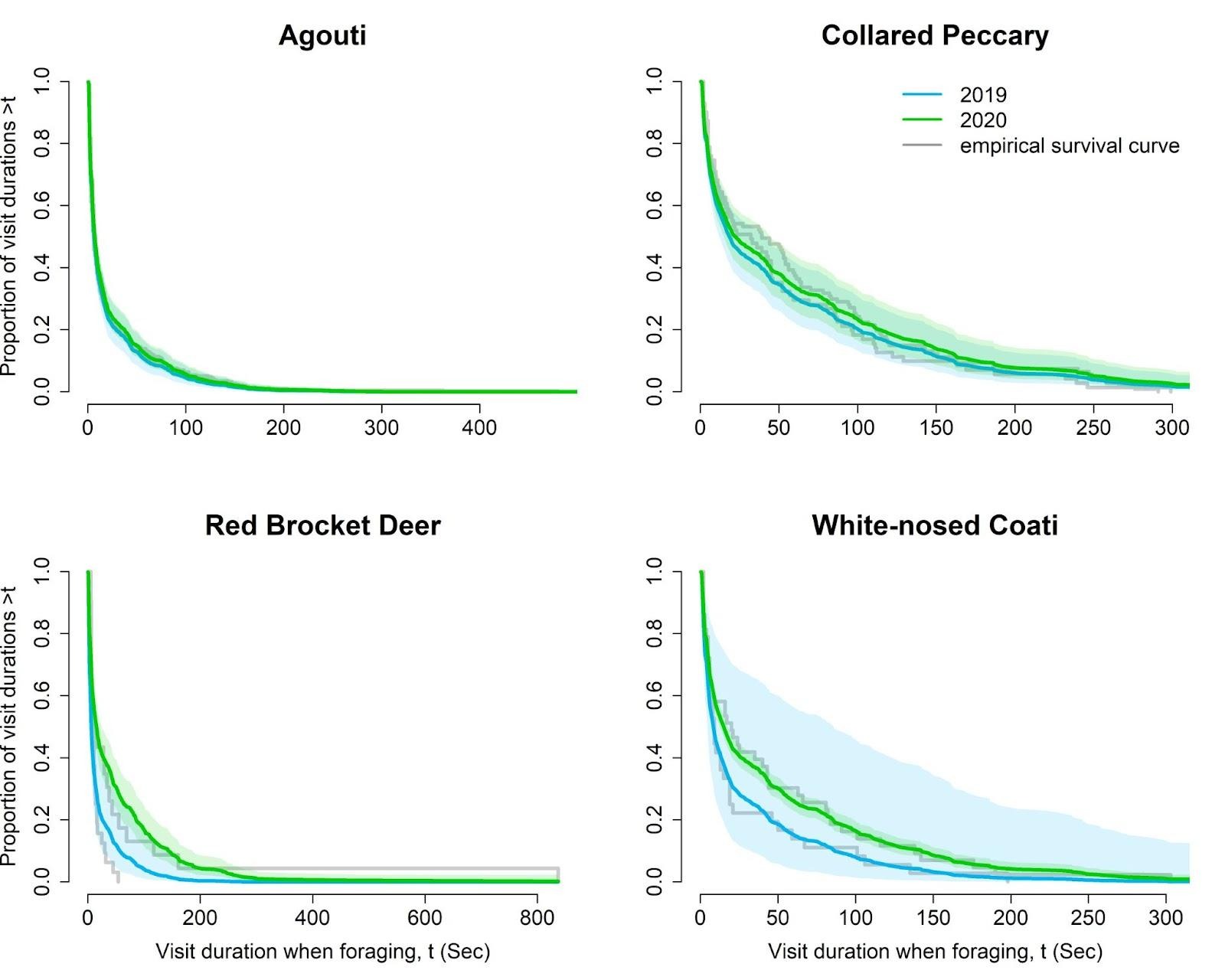


Supplementary Fig. 1: Cox Proportional Hazards model predictions for the visit duration when engaged in foraging behaviors for four mammal species (agouti, peccary, brocket deer and coati), comparing non-lockdown (blue) and lockdown (green). Grey curve represents the empirical Kaplan-Meier estimate curve. Shaded regions correspond to 95% confidence intervals.

Supplementary Table 1

Data on BCI from the physical long-term monitoring program (Paton, 2026) showed relatively minimal interannual variation in precipitation, humidity, solar radiation and temperature. This table shows differences between 2020 and 2019 (2020-2019) of four physical variables, where for example -10.2 refers to a decrease in 2020 in relation to 2019, and +20.3 refers to an increase.

|  | April | May | June | July |
| --- | --- | --- | --- | --- |
| Monthly rain (mm) | -10.2 | +20.3 | +60.5 | +83.3 |
| Humidity (%) | +3.6 | +1.4 | +1.5 | +1.7 |
| Solar radiation (w/m^2^) | -1.8 | +0.9 | -1.3 | -0.6 |
| Temperature (Celsius) | -0.1 | +0.3 | -0.6 | -0.3 |

Steven Paton. (2026). *Monthly Summary_BCI, horizontal*. Smithsonian Research Data Repository. [doi:10.60635/C3992K](https://doi.org/10.60635/C3992K), version: [doi:10.60635/C3HW5F](https://doi.org/10.60635/C3HW5F).

Supplementary Table 2

Mean and range (min - max) of empirical monthly *rate of events* for all species

|  | *Empirical estimates* | |
| --- | --- | --- |
| Species | *Non-lockdown* | *Lockdown* |
| *Humans* | 4.7 (0.2 – 27.3) | 0.5 (0 – 4.4) |
| *Agouti* | 61.2 (5.1 – 123.1) | 59.1 (9.8 – 144.6) |
| *Collared-peccary* | 12.6 (0.5 – 55.9) | 16.9 (0.6 – 59.5) |
| *Brocket deer* | 5.7 (0.3 – 23) | 6 (0.2 – 17.2) |
| *White-nosed coati* | 4.7 (0.5 – 9.9) | 0.3 (0 – 1.2) |
| *Ocelot* | 4.1 (0.7 – 17.5) | 3.5 (0 – 24.3) |
| *Paca* | 2.8 (0 – 11.1) | 3.2 (0 – 17. 7) |
| *Common opossum* | 1 (0 – 4.9) | 0.8 (0 – 4.7) |
| *Red-tailed squirrel* | 0.4 (0 – 2) | 0.6 (0 – 1.5) |
| *White-faced capuchin* | 0.2 (0 – 1.7) | 0.3 (0 – 1.2) |
| *Spiny rat* | 0.2 (0 – 1.8) | 0.2 (0 – 1.2) |
| *Baird’s tapir* | 0.1 (0 – 0.7) | 0.2 (0 – 2.5) |
| *Jaguarundi* | 0.2 (0 – 1) | 0 (0 – 0.2) |
| *Northern tamandua* | 0.1 (0 – 0.5) | 0.1 (0 – 0.5) |
| *Spiny pocket mouse* | 0 (0 – 0.7) | 0.1 (0 – 0.5) |
